# Research on Network Intrusion Detection Based on Weighted Histogram Algorithm for In-Vehicle Ethernet

**DOI:** 10.3390/s25113541

**Published:** 2025-06-04

**Authors:** Yutong Wang, Yujing Wu, Yihu Xu, Kaihang Zhang, Yinan Xu

**Affiliations:** College of Engineering, Yanbian University, Yanji 133002, China; 2023050056@ybu.edu.cn (Y.W.); yjwu@ybu.edu.cn (Y.W.); xuyh@ybu.edu.cn (Y.X.); 2689332993@ybu.edu.cn (K.Z.)

**Keywords:** In-Vehicle Ethernet, network security, intrusion detection, Audio Video Transport Protocol, weighted histogram algorithm

## Abstract

The Internet of Vehicles plays a crucial role in advancing intelligent transportation systems, with In-Vehicle Ethernet serving as the fundamental backbone network of the new generation of in-vehicle communication. However, In-Vehicle Ethernet faces various network security threats, including data theft, data tampering, and malicious attacks. This study focuses on network intrusion and security issues in In-Vehicle Ethernet, by analyzing the data characteristics of Audio Video Transport Protocol and potential network attack means. We innovatively propose a network intrusion detection method based on a weighted histogram algorithm. This method aims to enhance the security of In-Vehicle Ethernet. Experimental results show that the anomaly detection rate of the proposed weighted histogram algorithm in this study is 99.7%, which shows an improvement of 15.8% compared with the traditional Bayesian algorithm, and 6.9% higher than the decision tree algorithm. Thus, our approach enhances the stability and anti-attack ability of In-Vehicle Ethernet, providing a solid network security for In-Vehicle Networks.

## 1. Introduction

With the rapid development of Intelligent Connected Vehicles (ICVs), in order to satisfy the demand for large-scale data with low latency, high speed rate in In-Vehicle Networks (IVNs), in-vehicle communication architectures have become complex and diversified. ICVs utilize sensors such as radar, cameras, and Light Detection and Ranging (LiDAR) to detect the environment around the vehicle in real-time. Electronic Control Units (ECUs) generate control commands to establish stable data communication links with external devices like smartphones [1]. While improving the perception accuracy and real-time decision making of ICV, the network security issues are becoming increasingly serious [2]. Network attacks, data breaches, and malicious intrusions not only compromise the integrity and confidentiality of in-vehicle data but also endanger the functional safety of vehicles, user privacy, as well as life safety. Therefore, effective detection of network attacks is a core aspect of in-vehicle security.

In-Vehicle Ethernet supports the data transmission rate ranging from 100 Mbps to 1 Gbps and even higher. In-Vehicle Ethernet is a mainstream technology due to its high bandwidth, low latency, and secure communication characteristics in IVNs. In-Vehicle Ethernet meets the data transmission requirements of Advanced Driver Assistance Systems (ADASs) and In-Vehicle Infotainment (IVI). In-Vehicle Ethernet introduces a series of protocol standards, including IEEE 1722 Audio Video Transport Protocol (AVTP) [3], IEEE 802.1AS Generalized Precision Time Protocol (gPTP) [4], and IEEE 802.1Qat Stream Reservation Protocol (SRP) [5], currently integrated into IEEE 802.1Q protocol standard, it is used to define traffic shaping and scheduling in Time-Sensitive Networking (TSN). Through collaborative work between protocols, efficient data transmission and optimized network resources management are achieved. However, high bandwidth, high real-time performance, and open architecture of In-Vehicle Ethernet bring new network security risks. Developing efficient and reliable Intrusion Detection Systems (IDS) has become a critical research direction [6].

IDS enables real-time monitoring of network traffic and identifies potential security risks. Traditional network intrusion detection methods are primarily techniques based on statistical features, rules, and deep learning [7]. Wang et al. evaluated the applicability of 10 representative deep learning methods including ResNet model and Transfer model for IVNs intrusion detection. The experimental results demonstrated that deep learning-based intrusion detection methods could not identify unknown attacks, which limits the practical application in in-vehicle environments [8]. Almehdhar et al. classified IVNs intrusion detection methods into three categories: traditional machine learning techniques, deep learning techniques, and hybrid techniques. Almehdhar et al. emphasized the shift in intrusion detection from traditional based on signature to anomaly based detection methods. Future research needs in-depth exploration and improvement in computational efficiency, dataset size, cross-vehicle, and protocol generalization aspects [9]. In addition, Muslam, M.M.A. analyzed authentication mechanisms including two-factor authentication and Message Authentication Code (MAC), encryption algorithms such as asymmetric encryption, elliptic curve cryptography, and studied in-vehicle Public Key Infrastructure (PKI) key management protocols. Muslam, M.M.A. discussed the advantages and disadvantages of cryptographic techniques regarding security, efficiency, and computational overhead. Experimental results show that cryptographic techniques ensure the data transmission confidentiality and integrity, but challenges still exist in meeting real-time transmission requirements and managing computational complexity [10].

Although existing research has made a number of advances in in-vehicle intrusion detection, there are still deficiencies and research gaps. Most deep learning methods rely heavily on large-scale, high-quality labeled datasets to achieve higher detection accuracy. However, in the actual in-vehicle environments, sample acquisition is limited, and the data distribution is often uneven and unbalanced. Many existing intrusion detection methods have strong detection performance but high computational complexity, which makes it difficult to meet the stringent requirements of real-time and resource constraints in In-Vehicle Ethernet. In addition, the existing research mainly focuses on the traditional LIN and CAN buses, while the research on intrusion detection for AVTP of In-Vehicle Ethernet is still relatively weak; the related research results are scarce and still need further exploration.

Traditional network security mechanisms are difficult to meet the real-time requirements of In-Vehicle Ethernet AVTP. Improving IDS performance and real-time information processing capability are the key to guaranteeing network security. This study focuses on intrusion detection technology for In-Vehicle Ethernet AVTP, proposes an innovative intrusion detection method based on a weighted histogram algorithm, and adjusts the algorithm parameters by combining with dynamic optimization ideas. The approach improves detection accuracy and efficiency of abnormal data.

The rest of this article is organized as follows: Section 2 analyzes the data frame format and network attack types of AVTP for In-Vehicle Ethernet. Section 3 describes the weighted histogram algorithm in detail. Section 4 conducts experimental validation using the In-Vehicle Ethernet intrusion detection dataset and evaluates the performance of the algorithm. Section 5 summarizes the research and draws conclusions.

## 2. AVTP and Security Analysis of In-Vehicle Ethernet

While ICVs have significantly advanced the driving experience, they have also introduced critical challenges, including increased exposure to hacker attacks and system vulnerabilities. As a result, conventional in-vehicle bus networks can no longer be regarded as isolated and inherently secure systems. In-Vehicle Ethernet AVTP supports multiple Audio and Video (AV) formats. AVTP is widely used in diverse scenes. ICVs through integrate multimodal sensors provide comprehensive environment sensing capabilities for in-vehicle systems, enabling key functions such as environment prediction, collision warning, and path planning of vehicles [11]. Due to the openness and complexity of In-Vehicle Ethernet AVTP, it faces network security hazards related to data loss, network delay, and network intrusion during data transmission. In order to ensure the safe operation of vehicles, the systems must maintain critical data frames communication, reduce packet loss, and decrease information delay, thereby improving the safety and reliability of in-vehicle bus networks [12,13].

### 2.1. Introduction to In-Vehicle Ethernet AVTP

In-Vehicle Ethernet AVTP is the core protocol for AV data transmission. AVTP mandates 100 Mbps full-duplex Ethernet operation mode to meet the stringent communication requirements of ADAS and IVI for high quality, low latency. By incorporating timestamps and Stream IDentifier (Stream ID) mechanisms, AVTP achieves efficient management of multimedia streams. AVTP provides an important support for Quality of Service (QoS) in In-Vehicle Ethernet [14]. AVTP is based on TSN standards to orchestrate traffic scheduling and clock synchronization, ensuring the real-time and reliability of AV data transmission [15,16]. Furthermore, AVTP is based on the underlying Ethernet protocols such as IEEE 802.3, cooperates with the upper layer SOME/IP, and other application protocols. AVTP achieves the encapsulation, transmission, and control of AV data with high efficiency. These protocols jointly construct the multilayer communication architecture of In-Vehicle Ethernet.

In-Vehicle Ethernet frames with AV data operate at the data link layer of the Open Systems Interconnection Model (OSI). AVTP transmits data content, including media streams and control information. AVTP data frame consists of a header, destination MAC address, source MAC address, 802.1Q header, Ether-type field, AVTP packet, and Cyclic Redundancy Check/Frame Check Sequence (CRC/FCS). The AVTP packet includes a header, Stream ID, presentation time, format, packet info, and AV data. Among them, the header defines the type and sequence number of AV data, Stream ID identifies the data name. The presentation time indicates when the listener should submit AV data to the application layer for processing at a specific moment.

**Figure 1:**
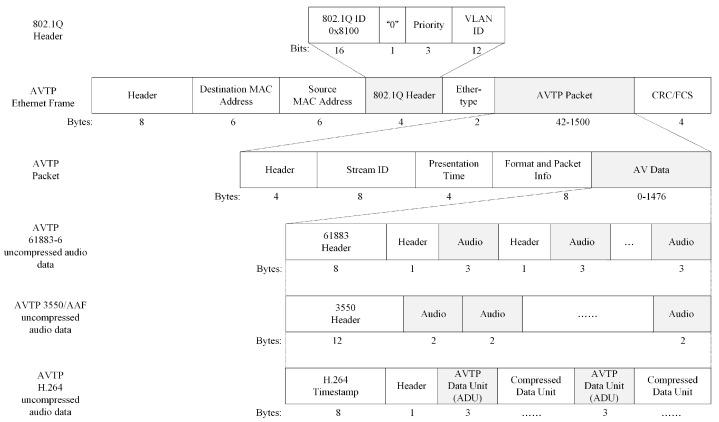
AVTP data frame format.

In the AV Data field, AVTP supports multiple uncompressed AV data formats, such as 61883-6, 3550/AAF, and AVTP H.264. Different data formats have different header structures and applicable standards. AVTP 61883-6 data format is typically used for audio transmission in IVI systems. AVTP 3550/AAF data format is used in the field of audio processing to deal with high resolution data. The AVTP H.264 data format supports synchronization processing for AV data. Figure 1 shows the data frame format of In-Vehicle Ethernet AVTP.

The data transmission of AVTP depends on the effective interaction between the talker and listener. To enable proper communication, both the talker and listener must be located within the same Virtual Local Area Network (VLAN). The talker is responsible for generating AV data and encapsulating the data in AVTP packets. Each AVTP packet contains AV data, presentation time, and other control information. This information, through In-Vehicle Ethernet, is sent to the listener. During data transmission, the packets are priority queued and traffic-shaped based on the IEEE 802.1Qav protocol to ensure that AVTP packets can be preferentially transmitted. After receiving AVTP packets, the listener first parses the packet header information and extracts the payload data, the IEEE 802.1AS protocol is used to synchronize the playback of AV data [17].

### 2.2. Network Security Analysis of In-Vehicle Ethernet

The open architecture of In-Vehicle Ethernet triggers network security issues. Due to the absence of perfect encryption and authentication mechanisms in the network security protection measures, In-Vehicle Ethernet faces security risks. These security issues seriously threaten vehicle safety and user privacy [18]. Attackers can compromise IVNs through physical access and remote exploitation, posing a significant threat to in-vehicle communication systems based on Ethernet architecture. Common entry points include the On Board Diagnostics (OBD) port, Bluetooth/Wireless Fidelity (WiFi) interfaces within IVI, telematics unit systems, and other externally facing components susceptible to weak credentials or firmware vulnerabilities. Once network access is achieved, attackers leverage these interfaces as entry points to craft diverse attack vectors, targeting communication protocols, data integrity, and transmission mechanisms within the In-Vehicle Ethernet system. Attackers use both passive and active methods to attack In-Vehicle Ethernet, enabling data interception, traffic manipulation, and service disruption.

A passive attack represents an attack method that does not actively interfere with the data communication process. Due to the fact that attackers do not directly modify data frames, but rather through a passive attack on data monitoring, attackers can achieve more complex active attacks. After accessing the In-Vehicle Ethernet, attackers obtain information such as vehicle operating status, network topology, identifiers of AV service streams (Stream ID), and communication cycles by monitoring the communication traffic between target ECUs. Regarding AVTP of In-Vehicle Ethernet, attackers analyze the temporal characteristics of AVTP packets, such as timestamps, transmission cycles, and arrival intervals, to speculate the activity status of AV data and provide preparation information for subsequent active attacks.

Common types of active attacks in In-Vehicle Ethernet include Denial of Service (DoS) attack, injection attack, and replay attack [19]. The DoS attack is the most serious network attack, as it injects a large amount of invalid data into ECUs, overwriting the original valid data. Authorized users are unable to access resources normally, resulting in communication interruption. DoS attacks not only consume excessive network bandwidth and resources but also lead to data frame loss. Andreica et al. conducted penetration testing for DoS attacks on in-vehicle navigation systems, using the hping3 tool in Kali Linux to launch an attack on User Datagram Protocol (UDP) port 26121, and redirecting network traffic to the local network port 26015. The results demonstrated that in-vehicle navigation systems failed to receive data properly under the DoS attack. To cope with the DoS attack, Andreica et al. suggested the use of the Transmission Control Protocol (TCP) instead of UDP, incorporating asymmetric/symmetric encryption algorithms, and optimizing remote firmware update mechanisms to enhance the network security [20]. The DoS attack of In-Vehicle Ethernet AVTP can be implemented through remote intrusion into the vehicle gateway or by physically implanting malicious devices. Attackers use previously collected protocol feature information to forge a large number of erroneous AV data frames, which carry illegal VLAN tags or unreasonable Priority Code Point (PCP) values. And inject them into the network at high frequencies, thereby covering or blocking normal data transmissions. The listener cannot receive legitimate AV data, and the correct data are overwritten. Due to communication relying on delay sensitive queue services mechanisms, once the normal data are disrupted, it directly causes interruption and significant delay in IVI, ultimately impairing users’ perception and judgment of vehicle condition.

An injection attack exploits the information of data structures and communication protocols to inject forged data into the network, tricking vehicles into executing false control commands. Feng et al. established a threat model for injection attack based on Connected Vehicle-based Traffic Signal Control (CV-TSC) systems, using an On Board Unit (OBU) to obtain speed and position from Basic Safety Messages (BSMs), Signal Phase and Timing (SPaT) information broadcast in the network. Feng et al. extracted and fabricated key traffic features of the information to carry out an injection attack [21]. Based on an in-depth understanding of in-vehicle systems and communication protocols, attackers through maliciously flashed ECUs and external diagnostic tools to connect to the network to achieve an injection attack. Design and fabricate legitimate data frames carrying specific control commands and configuration instructions. These forged frames can simulate normal AV data frames. Malicious nodes inject the altered commands for video stream control, audio switching, image termination, and other operational control information into IVNs, so that the listener can mistake them for legitimate data and realize an injection attack. An injection attack leads to AV data interruptions and delays. Attackers can induce the in-vehicle systems to perform unauthorized operations, such as forcibly turning off camera images, switching voice prompts, or pausing reverse image playback.

Replay attack exploits the lack of timestamp authentication mechanisms in In-Vehicle Ethernet protocols. The attack involves intercepting valid data transmitted in the network, tricking vehicles into performing an illegal operation by delaying the transmission and duplicating the forwarding. Abu Al-Haija et al. utilized the vulnerability of keyless entry systems in vehicles to simulate replay attack scenarios. By intercepting the transmitted data, duplicating the transmitted data, and resending the transmitted data to the vehicle receiver. In-vehicle systems are tricked into misinterpreting the request as legitimate, leading to unauthorized unlocking [22]. The real case fully confirms the feasibility of a replay attack. Video footage released by the British police shows that hackers used two relay devices to successfully infiltrate the vehicle keyless entry and start systems within 1 min, completing a remote theft [23]. The replay attack of In-Vehicle Ethernet AVTP is caused by a passive attack, which represents a typical active attack method based on temporal manipulation and is usually implemented during vehicle operation. After gaining access to the IVNs, the attacker first passively monitors AV traffic data frames and retransmits the captured critical AV content to the In-Vehicle Ethernet listener at a specific moment. Owing to the lack of strong encryption and authentication mechanisms in AVTP, the listener cannot distinguish between authentic and replayed content, thereby disrupting the transmission of AV data, triggering video frame rollback, repeated playback of voice navigation information, and abnormal system function response. Replay attacks result in the replay of vehicle video frames and voice navigation information, which causes interference to users. Replay attacks often do not rely on system vulnerabilities and only require intrusion privileges at the data link layer, making them highly feasible for implementation. This study uses a real public dataset for intrusion detection of replay attacks within In-Vehicle Ethernet AVTP.

During the data transmission process of AVTP, the talker and listener become potential targets for hacker attacks. da Luz et al. described the process of hacker attacks. Hackers infiltrate the talker through network intrusion to carry out attacks, disrupting the normal reception and processing of data properly at the listener side. Hacker attacks destroy the integrity of the communication process, triggering errors in vehicle decision-making systems, forcing vehicles to perform dangerous operations such as stopping the engine, collision, and rear-end crashes [24]. In addition, vehicles connect to external network systems through external network interfaces like WiFi and Bluetooth. Hackers use these network interfaces to steal vehicles’ location information, AV information, and user privacy information. Such breaches enable remote control, information theft, and tampering with control commands, thereby executing malicious network attacks [25].

To address the network security threats faced by In-Vehicle Ethernet, three main solutions are currently employed, including data protection based on cryptographic technologies, network access control utilizing firewall technologies, and real-time monitoring through intrusion detection technologies, thus ensuring the network security of In-Vehicle Ethernet [26].

Cryptographic technologies enhance the protection against network attacks by establishing a perfect key management system for In-Vehicle Ethernet, combined with advanced encryption algorithms and authentication mechanisms. Zhu et al. first proposed and evaluated a post-quantum enhanced session key negotiation process based on the NTRUEncrypt algorithm for In-Vehicle Ethernet context information. The experimental results shown that the NTRUEncrypt algorithm is 66.06 times faster than the Elliptic Curve Diffie–Hellman (ECDH) algorithm, and 1530.98 times faster than the Rivest–Shamir–Adleman (RSA) algorithm in terms of execution speed. The NTRUEncrypt algorithm provides an efficient and secure session key negotiation scheme for In-Vehicle Ethernet to strengthen the confidentiality of in-vehicle communication [27]. Chen et al. proposed an In-Vehicle Ethernet key management system. By randomly generating keys, combining elliptic curve encryption and the Schnorr signature algorithm to complete the key distribution. Encryption and authentication of multimedia data are realized using AES-128 encryption algorithm and HMAC algorithm, which solves the problem of real-time transmission and the network security of In-Vehicle Ethernet [28].

Firewall technologies effectively identify and block abnormal behaviors in the network based on predefined security rules and access control rules. Luo et al. analyzed vehicle security risks and proposed a firewall-based network security architecture based on firewall technologies. Two independent firewall systems were designed for Flexible Data-Rate (CAN/FD) network and In-Vehicle Ethernet, respectively. The CAN/FD network used the packet filtering mechanism to monitor network traffic, and the In-Vehicle Ethernet used the Stateful Packet Filter (SPF) mechanism to implement the firewall function, which constructed a multilevel IVNs security protection system [29].

Compared to passive defense mechanisms represented by cryptography and firewall technologies, intrusion detection technologies enable real-time data traffic monitoring, with proactively trigger response mechanisms upon detection of anomalous behaviors. Alkhatib et al. conducted a comprehensive evaluation of neural networks with various unsupervised deep learning models, such as autoencoder, convolutional autoencoder, and traditional machine learning models in detecting abnormal traffic using a real In-Vehicle Ethernet AVTP dataset. The experimental results show that under diverse evaluation scenarios, unsupervised deep learning models significantly outperform other traditional anomaly detection models [30]. Han et al. combined wavelet transforms with a deep convolutional neural network to propose an efficient method for anomaly detection in In-Vehicle Ethernet, balancing detection accuracy and efficiency [31]. Additionally, in the face of complex driving environments, the perception area of vehicles is affected by factors such as occlusion, overlap, and adverse weather, which leads to a decrease in the sensing ability of vehicle sensors and a reduction in their sensitivity. Wang et al. analyzed detection algorithms, sensor characteristics, and application scenarios for various sensors in vehicles. As a result, the inability of a single sensor to comprehensively perceive the dynamically changing environment in vehicle detection, a deep learning fusion algorithm is used to effectively integrate the sensing information of multiple sensors. They combined the advantages of high-resolution images from cameras, precise measurement from millimeter wave radars, and three-dimensional point cloud processing from LiDAR, to enhance the detection accuracy and stability of vehicles in complex environments [32].

To safeguard the confidentiality and reliability of the data transmission process in In-Vehicle Ethernet, it is essential to conduct a rigorous analysis of potential attack vectors and security protection systems. Designing high-performance IDS alongside a corresponding defense architecture is critical to comprehensively improving the network’s resilience against sophisticated threats.

## 3. Intrusion Detection Method Based on Weighted Histogram Algorithm

**Figure 2:**
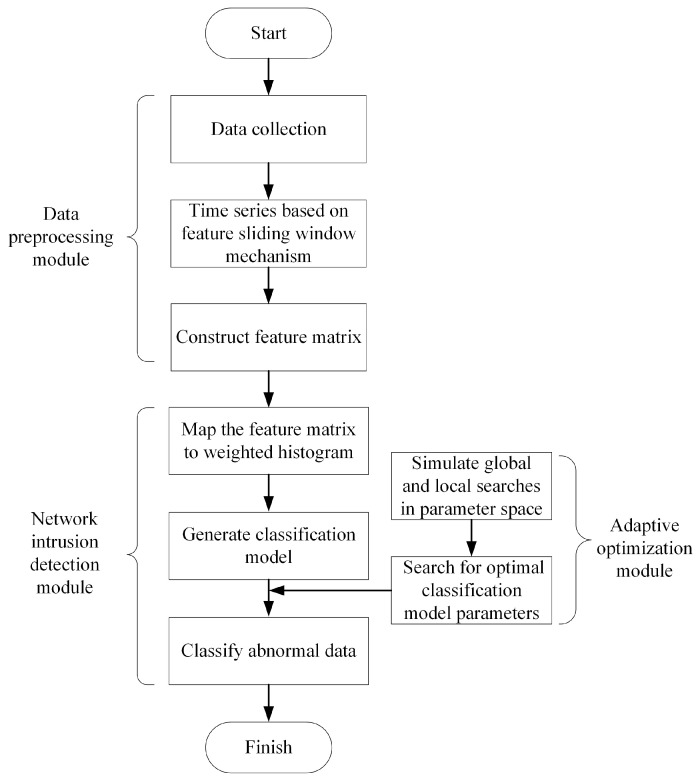
Network intrusion detection architecture.

To further enhance the network security performance of In-Vehicle Ethernet, this study proposes an intrusion detection method based on a weighted histogram algorithm. The proposed method was developed specifically for the characteristics of AV data frames in IVNs. The network intrusion detection architecture of In-Vehicle Ethernet is shown in Figure 2. The network intrusion detection architecture consists of a data preprocessing module, a network intrusion detection module, and an adaptive optimization module. By strengthening the ability to identify abnormal AV data, the architecture offers an efficient and reliable solution for securing in-vehicle communication networks.

### 3.1. Data Preprocessing Module

In the data frame for AVTP of In-Vehicle Ethernet, each AVTP packet has 438 bytes, and the value of each byte is an integer value between 0 and 255. Jeong et al. analyzed the byte-level distribution of 100 consecutive AVTP packets by visualization and found that the first 58 bytes have obvious structural regularity and temporal order [33]. Except for the Header and payload field parts, the majority of AVTP header values remained constant. These static characteristics are particularly susceptible to disruption during abnormal behaviors. The payload section after 59 bytes lacks statistical structure and has high entropy. Especially in the case of the replay attack, repeated patterns become visually prominent in both the header and the initial segment of the payload. This regularity provides a robust basis for efficient anomaly detection. To satisfy the stringent real-time communication requirements while ensuring efficient anomaly detection of In-Vehicle Ethernet, this study focuses on analyzing the first 58 bytes of each AVTP packet. This approach enables the system to effectively monitor network traffic and detect data anomalies while significantly reducing computational complexity.

Building upon these insights, this study focuses on the feature extraction strategy of the first 58 bytes, significantly reducing computational overhead while maintaining high detection accuracy against replay attack-based intrusion.

**Figure 3:**
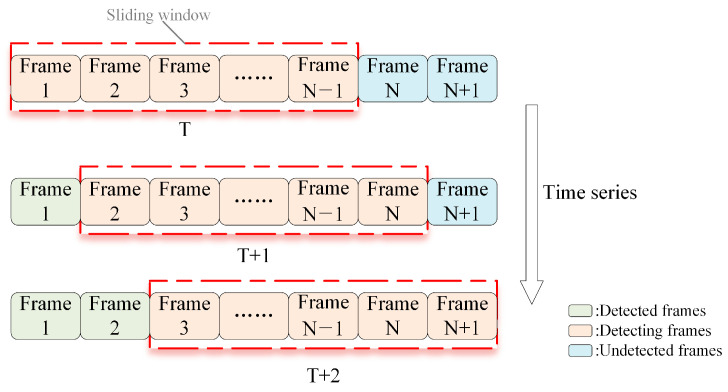
Based on a feature sliding window mechanism.

This article is based on the feature sliding window mechanism to represent time series, as illustrated in Figure 3. The sliding window mechanism dynamically updates data to achieve a smooth connection between data. The sliding window mechanism can maintain contextual continuity over short intervals. As new data enter the sliding window, dynamic network intrusion detection is performed according to the updated window data.

N is the total number of data frames. Extract the first 58 bytes from each frame $\left(\mathrm{Frame}1,\;\mathrm{Frame}2,\;\ldots ,\;\mathrm{FrameN}\right)$ to construct a feature matrix X, as shown in Equation (1).

(1) $$X=\left[\begin{array}{ccc} x_{1,1} & \ldots & x_{1,58}\\ \vdots & \ddots & \vdots \\ x_{\omega ,1} & \ldots & x_{\omega ,58} \end{array}\right]$$

In this matrix, each row corresponds to the sliding window size ω, and each column represents a specific feature value from the first 58 bytes of each data frame. The element $x_{i,j}$ denotes the value at the i-th row and j-th column. The feature vector of the i-th frame is $X_{i}=\left[x_{i,1}{,\;x}_{i,2}{,\;x}_{i,3}{,\;\ldots \ldots ,\;x}_{i,58}\right]$.

This study is entirely focused on the data of AVTP for In-Vehicle Ethernet. The data preprocessing module selects the first 58 bytes, uses a sliding window for temporal context, and constructs a feature matrix X for the subsequent network intrusion detection module.

### 3.2. Network Intrusion Detection Module

**Figure 4:**
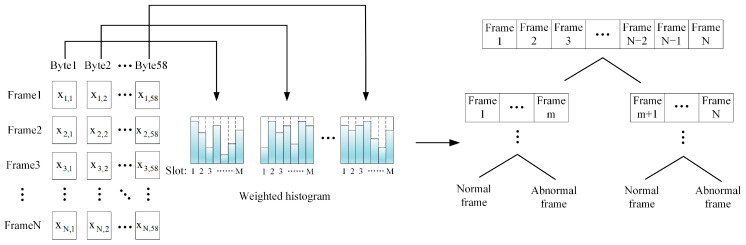
Network intrusion detection module architecture.

In the network intrusion detection module, a map of the feature values of each byte is used to create a weighted histogram. By calculating the classification gain, the optimal classification point is determined in the classification model to achieve efficient network intrusion detection. Figure 4 shows the architecture of the network intrusion detection module based on the weighted histogram algorithm.

For each of the 58 bytes in a data frame, 58 weighted histograms are constructed separately. Each weighted histogram contains one byte of feature values. The set of feature values at the j-th column is $\left\{x_{1,j}{,x}_{2,j}{,x}_{3,j}{,\ldots \ldots ,x}_{N,j}\right\}$. Divide each weighted histogram into M slots, with the width of each slot Δ, as shown in Equation (2). The feature values of each byte are mapped to the corresponding ${\mathrm{Slot}(x}_{i,j}),$ according to Equation (3), ${\max(x}_{i,j})$ and ${\min(x}_{i,j})$ denote the maximum and minimum values of each byte feature.

(2) $$\Delta =\frac{\max(x_{i,j})-\min(x_{i,j})}{M}$$

(3) $$\begin{array}{c} Slot(x_{i,j})=\frac{x_{i,j}-\min(x_{i,j})}{\Delta }\\ \left(1\leq i\leq 58,1\leq j\leq N\right) \end{array}$$

The distance $d_{x_{i,j}{,x}_{i,j}^{\prime }}$ between the data frame $x_{i,j}$ and its neighboring data frame $x_{i,j}^{\prime }$ is calculated by Equation (4), and sorted according to the distance size.

(4) $$d_{x_{i,j},x_{i,j}^{\prime }}=\sqrt{\sum_{j=1}^{58}{\left(x_{i,j}-x_{i,j}^{\prime }\right)}^{2}}$$

By utilizing the n nearest data frames with the smallest distance from the data frame $x_{i,j}$, calculate the average distance $d_{i,j}^{\mathrm{avg}}$, as shown in Equation (5). Since the average distance between abnormal data frames and neighboring data frames is greater than the distance between normal data frames. The average distance of data frames can reflect the similarity and difference between data frames and neighboring data frames.

(5) $$d_{i,j}^{avg}=\frac{1}{n}\sum_{S_{i,j}}d_{x_{i,j},x_{i,j}^{’}}$$

In order to highlight the abnormal data, this study adopts a weight assignment strategy based on the average distance, and assigns a corresponding weight $\omega_{i,j}$ to each data frame, as shown in Equation (6). The weight reflects the degree of abnormality in the data frame, with larger average distances corresponding to smaller weights.

(6) $$\omega_{i,j}=\frac{1}{d_{i,j}^{avg}}$$

Normalize the average distance in Equation (5) to obtain the target value $y_{i,j}$ of the data frame, as shown in Equation (7). The target value is used to measure the degree of deviation between data frames and neighboring data frames, thus facilitating the identification of abnormal data frames. $d_{\max}^{\mathrm{avg}}$ and $d_{\min}^{\mathrm{avg}}$ represent the maximum and minimum average distance across all data frames, the target value range is between [0, 1] after the normalization process.

(7) $$y_{i,j}=\frac{d_{\max}^{avg}-d_{i,j}^{avg}}{d_{\max}^{avg}-d_{\min}^{avg}}$$

The weighted mean is calculated using the weight and target value to derive the initial prediction value $F_{0}$, as shown in Equation (8). The initial prediction value serves as the baseline for the update iteration, providing a reasonable starting point for the iterative process.

(8) $$F_{0}=\frac{\sum_{i=1}^{N}\omega_{i,j}y_{i,j}}{\sum_{i=1}^{N}\omega_{i,j}}$$

In the iterative process, k is the number of iterations. Calculate the residual $r_{k}$ between the target value and the current prediction value $F_{k}$. The residual effectively identifies abnormal data frames, as shown in Equation (9).

(9) $$r_{k}=y_{i,j}-F_{k}$$

Based on the current prediction value and current residual, the next round prediction value $F_{k+1}$ is updated using Equation (10). Through constantly updating the iterative prediction value, the residual difference between the target value and the prediction value is reduced, gradually bringing the prediction value closer to the target value.

(10) $$F_{k+1}=F_{k}+\sum_{i=1}^{N}\omega_{i,j}\cdot r_{k}$$

Calculate the Weighted Mean Squared Error (WMSE) loss function ${L(y}_{i,j}{,F}_{k})$ of each data frame, as shown in Equation (11). The loss function reflects the gap between the current prediction value and the target value according to the weight of different data frames.

(11) $$L\left(y_{i,j},F_{k}\right)=\frac{1}{\sum_{i=1}^{N}\omega_{i,j}}\sum_{i=1}^{N}\left[\omega_{i,j}{\left(y_{i,j}-F_{k}\right)}^{2}\right]$$

Based on the loss function and current prediction value, calculate the first-order $g_{i,j}$ and second-order $h_{i,j}$ gradients of each data frame, as shown in Equations (12) and (13). The first-order gradient and second-order gradient represent the first-order and second-order derivatives of the loss function with respect to the prediction value, reflecting the rate at which the loss function changes relative to the current prediction value.

(12) $$g_{i,j}=\frac{\partial L(y_{i,j},F_{k})}{\partial F_{k}}=-2\omega_{i,j}\cdot r_{k}$$

(13) $$h_{i,j}=\frac{\partial^{2}L(y_{i,j},F_{k})}{\partial^{2}F_{k}}=2\omega_{i,j}$$

Aggregate the first-order and second-order gradients corresponding to all data frames within each slot, and the optimal classification point is determined based on the accumulated gradients of each slot. The gradients associated with the m-th slot are accumulated as shown in Equation (14). To ensure that each slot contributes to the split evaluation, traverse each slot sequentially from 1 to M and divide gradients into left and right subset sums $G_{m}^{\mathrm{Left}}$, $G_{m}^{\mathrm{Right}}$. Calculate the classification gain $G_{\mathrm{ain}}$ of each slot as shown in Equation (15). Select the data frame corresponding to the interval with the maximum classification gain as the optimal classification point in the classification model, so as to determine the classification scheme and complete the classification of normal and abnormal data frames.

(14) $$G_{m}=\sum_{x_{i,j}\in m}g_{i,j},H_{m}=\sum_{x_{i,j}\in m}h_{i,j}$$

(15) $$G_{ain}=\frac{1}{2}(\frac{{\left(G_{m}^{Left}\right)}^{2}}{H_{m}^{Left}+\lambda }+\frac{{\left(G_{m}^{Right}\right)}^{2}}{H_{m}^{Right}+\lambda }-\frac{G_{m}^{2}}{H_{m}+\lambda })(\lambda =0.001)$$

The network intrusion detection module introduces a weighted histogram algorithm designed for byte-level anomaly detection in AV data. The distance-based weighting, target value normalization, and iterative residual updating strategies form the core of this detection module.

### 3.3. Adaptive Optimization Module

**Figure 5:**
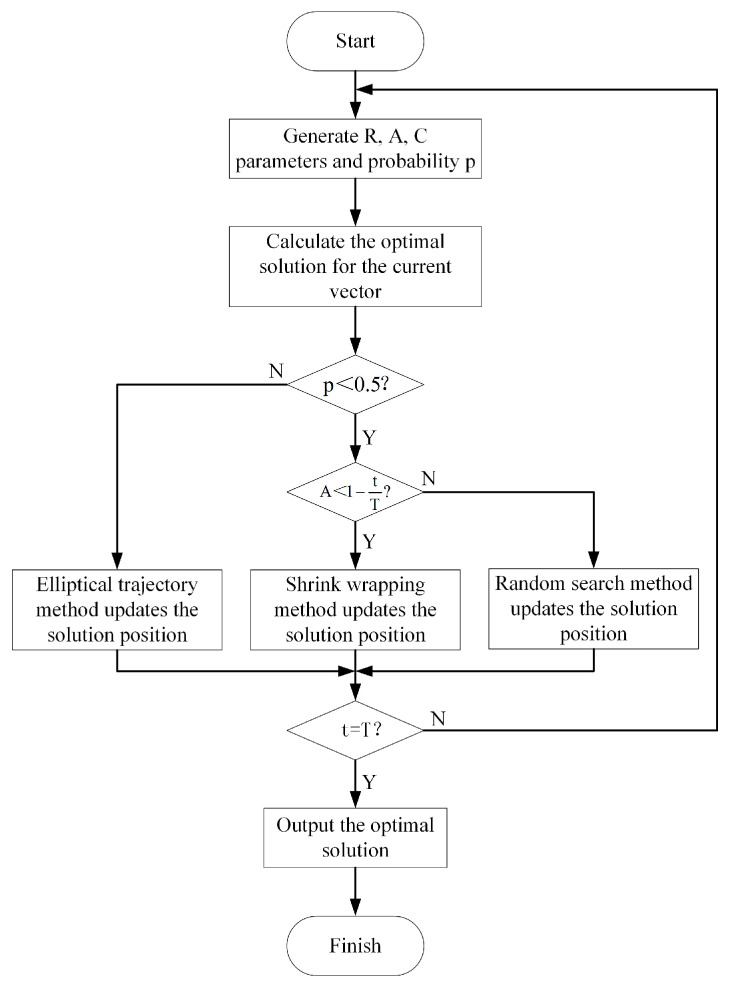
Adaptive optimization module flowchart.

To further enhance the accuracy of network intrusion detection, this study introduces an adaptive optimization module based on a weighted histogram algorithm. The adaptive optimization module performs global and local searches in the simulated parameter space to obtain the optimal solutions for three parameters: feature sampling rate $s_{1}$, classification node number $s_{2}$, and maximum classification frame rate $s_{3}$. The flowchart of the adaptive optimization module is shown in Figure 5.

Implement a dynamic update mechanism by defining three parameters R,A,C, as shown in Equations (16)–(18). Among them, parameter R is a random number generated by a random generator to randomize the search behavior, with a value range between [0, 1]. Parameter A regulates the size of the search range by controlling the expansion and contraction of the search area, t and T represent the current and maximum number of iterations. Parameter C determines the direction and position of the next update iteration, thereby adjusting the search method.

(16) R=rand[0,1]

(17) $$A=2\left(1-\frac{t}{T}\right)$$

(18) C=2R

The update method at the next moment is determined by introducing a random dynamic probability p, as shown in Equation (19). A value of p ≥ 0.5 triggers the global exploration stage, whereas p < 0.5 initiates the local development stage.

(19) p=rand[0,1]

In the initial stage, randomly generated F initial vectors are used as search starting points, and each vector contains three parameters, such as feature sampling rate, classification node number, and maximum classification frame rate, as shown in Equation (20). By calculating the loss function in Equation (11), the parameter combination corresponding to the minimum value of the loss function is the current optimal solution $S^{*}\left(t\right)$.

(20) $$S_{i}=\left[s_{i1},s_{i2},s_{i3}\right],i=1,2,\ldots ,F$$

During the global exploration stage, distance $D_{1}$ is calculated between the current solution S(t) and the current optimal solution $S^{*}\left(t\right)$. The position of the solution at the next moment S(t+1) is updated by employing an elliptical trajectory method, as shown in Equations (21) and (22). b is the elliptical parameter, and l is a random number between [−1, 1].

(21) $$D_{1}=\left|S^{*}\left(t\right)-S\left(t\right)\right|$$

(22) $$\begin{array}{c} S\left(t+1\right)=S^{*}\left(t\right)+e^{bl}\cos\left(2\pi l\right)\cdot D_{1}\\ \left(b=1.5,l=rand[-1,1]\right) \end{array}$$

During the local development stage, if $A\;<\;1-\frac{t}{T}$, calculate the distance $D_{2}$ between the current solution and the current optimal solution. The position of the next moment solution is updated using a shrink wrapping method, as shown in Equations (23) and (24). If $A\;\geq \;1-\frac{t}{T}$, in order to prevent falling into a local optimal solution, the position of the next moment solution is updated by using the random search method, thus enhancing the randomness and diversity of the search stage, as shown in Equations (25) and (26). Among them, $S_{\mathrm{rand}}\left(t\right)$ represents a random solution, $D_{3}$ is the distance between the random solution and the current solution.

(23) $$D_{2}=\left|C\cdot S^{*}\left(t\right)-S\left(t\right)\right|$$

(24) $$S\left(t+1\right)=S^{*}\left(t\right)-A\cdot D$$

(25) $$D_{3}=\left|C\cdot S_{rand}\left(t\right)-S\left(t\right)\right|$$

(26) $$S\left(t+1\right)=S_{rand}\left(t\right)-A\cdot D$$

In the initial stage of the adaptive optimization module, the value ranges of three parameters, feature sampling rate $s_{1}$, number of classification nodes $s_{2}$, and the maximum number of classification frames $s_{3}$, are firstly set as [0.5, 1], [10, 30], and [3, 10], respectively. Feature sampling rate $s_{1}$ initial range is set as [0.5, 1] to balance the detection accuracy and efficiency. Setting the lower limit to 0.5 ensures that at least half of the fields in the extracted 58 bytes are retained to participate in the calculation, which can speed up the processing speed while avoiding losing too much information and still capturing the main features. Setting the upper limit to 1 in cases where the abnormal features are scattered and the changes of individual fields are not obvious, the anomalies are missed if only a portion of the fields are sampled, so all of the fields need to be used to ensure that the anomalies can be captured. The number of classification nodes $s_{2}$ initial range is set as a compromise between the feature dimensions and the real-time requirements. If there are few classification nodes, the decisions can only be made based on a few data frames. However, if the number of nodes is set to a high level, although the accuracy is improved, the computational complexity will be greatly increased, and overfitting is prone to occur. The number of classification nodes $s_{2}$ is lower limit than 10, it is sufficient to cover the classification contour. When the upper limit is 30, it allows for more precise partitioning of data and adapts to complex anomaly detection requirements. The maximum number of classification frames $s_{3}$ is the maximum width of the sliding window. The initial range is set as [3,10], the minimum value can quickly detect sudden abnormal situations, and the maximum value can capture sufficient temporal context, striking a balance between maintaining continuity in temporal features and controlling the computational burden.

Through the repeated iteration process, the current solution and loss function are continuously updated, ultimately converging toward the optimal solution parameter combination that minimizes the loss function.

The adaptive optimization module employs an original search mechanism inspired by metaheuristics, designed to dynamically adjust key parameters in the network intrusion detection module. By integrating global elliptical search and local development search, this module enhances detection accuracy while combining the adaptive optimization module with the network intrusion detection module. This is an approach tailored specifically for real-time In-Vehicle Ethernet AV data.

## 4. Experimental Simulation and Result Analysis

CANoe tool is a bus development environment launched by Vector, a German (Stuttgart) company in 1996. CANoe tool is a simulation platform used for the design and performance analysis of bus networks, CANoe supports various in-vehicle communication networks such as LIN, CAN, FlexRay, In-Vehicle Ethernet, and so on. This study uses the CANoe-Ethernet tool to build an experimental simulation environment for the In-Vehicle Ethernet bus, and the experimental setup integrates software and hardware to validate the performance of the proposed algorithm.

**Figure 6:**
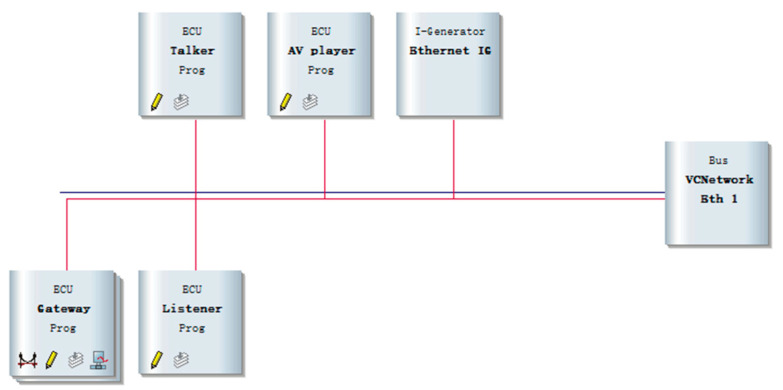
In-Vehicle Ethernet topology architecture.

**Figure 7:**
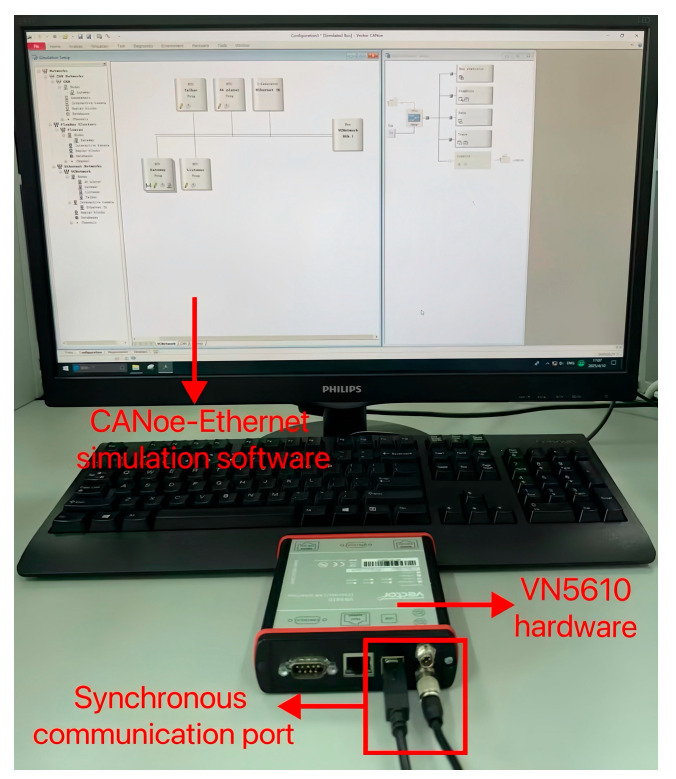
In-Vehicle Ethernet experimental simulation environment.

The network topology architecture of In-Vehicle Ethernet is shown in Figure 6. Gateway realizes the data transmission between different buses, the talker and listener simulate the data transmission, and the reception of AVTP. The AV player node is used to simulate playback media data, and the Ethernet Interactive Generator (Ethernet IG) node defines the sent data frame content and simulates the injection of abnormal data frames. All of the above nodes are interconnected through the In-Vehicle Ethernet network bus (Eth1). This study utilizes CANoe-Ethernet simulation software version 9.0 and the VN5610 hardware device as the Ethernet communication interface. VN5610 is connected to the PC through the synchronous communication port, with a combination of hardware and software to achieve efficient and stable communication. The in-vehicle Ethernet experimental simulation environment is shown in Figure 7.

This study employs an in-vehicle bus intrusion dataset publicly released by the research team at Korea University. Researchers captured real datasets through the BroadR-Reach testing platform [34]. The dataset is stored in PCAP file format and viewed using data analyzers such as Wireshark. Attackers inject AVTP Data Units (AVTPDUs) into In-Vehicle Ethernet, In-Vehicle Ethernet data packet carrying AV content. By rapidly reinserting a sequence of previously transmitted AVTPDUs, the attacker causes the listener playback end to erroneously output repeated video frames. In order to simulate the scenario of AVTP replay attacks in In-Vehicle Ethernet, the Korea University research team extracted 36 consecutive AVTPDUs from a real AVTP stream, sufficient to reconstruct a single video frame. The attacker repeatedly injected these 36 consecutive AVTPDUs into In-Vehicle Ethernet, simulating the effect of video frames being replayed. As shown in Table 1, a randomly obtained dataset (Randomly Obtain Dataset, ROD) comprises both all normal data and replay attack data, serving as the dataset for In-Vehicle Ethernet intrusion detection. The ROD data are allocated to the training and test sets according to the ratio of 3:7. For experimental purposes, the training set is used to undertake the process of intrusion detection, while the testing set is used for performance evaluation and result analysis. The label data (Label) specifically refers to the 36 consecutive AVTPDUs extracted from actual multimedia streams.

In order to verify the validity and reliability of the weighted histogram algorithm, this article evaluates four performance indicators, including True Positive (TP), True Negative (TN), False Positive (FP), and False Negative (FN). Among them, TP is the number of correctly predicted normal data frames, and TN denotes the number of abnormal data frames predicted as abnormal. FP refers to the number of abnormal data frames that are misjudged as normal, and FN indicates the number of normal data frames incorrectly classified as abnormal. Based on TP, TN, FP, and FN, calculate Accuracy, Precision, Recall, and F1_score of network intrusion detection.

Accuracy is the proportion of normal data frames classified to the total data frames, as shown in Equation (27).

(27) $$Accuracy=\frac{TP+TN}{TP+TN+FP+FN}$$

Precision is defined as the proportion of actual normal frames among the frames classified as normal, as shown in Equation (28).

(28) $$\mathrm{Pr}ecision=\frac{TP}{TP+FP}$$

Recall is the ratio of all normal data frames in the dataset that are classified correctly, as shown in Equation (29). A higher recall indicates a lower probability of normal data frames being misjudged as abnormal.

(29) Recall=TPTP+FN

The F1_score is the harmonic mean of precision and recall, as shown in Equation (30). The F1_score combines a balanced consideration of precision and recall, making it suitable for cases with an imbalanced ratio of normal and abnormal data frames.

(30) $$F1\_score=2\times \frac{precision\times recall}{precision+recall}$$

**Figure 8:**
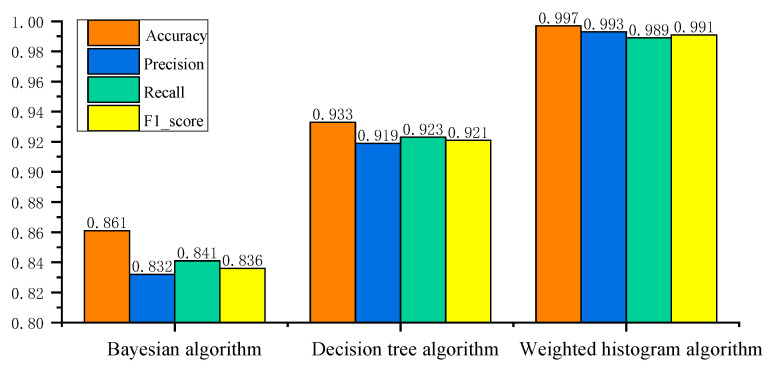
Comparison of network intrusion detection results.

A comparative analysis was conducted of the traditional Bayesian algorithm, the decision tree algorithm, and the weighted histogram algorithm proposed in this study. Figure 8 shows the network intrusion detection results of three algorithms, and Table 2 compares the network intrusion detection performance of three algorithms. Through the adaptive optimization module, obtain the optimal solution parameter combination for feature sampling rate $s_{1}$, classification node number $s_{2}$, and maximum classification frame rate $s_{3}$, as shown in Table 3.

The simulated search space is constructed with three value ranges, and actively explores the parameter combination, ultimately obtaining the optimal solution parameter combination is $S=\left[0.584,\;28,\;9\right]$, thereby achieving the efficient identification of abnormal data behavior.

From the experimental results analysis, the network intrusion detection performance varies significantly across the three evaluated algorithms. Four evaluation indicators (Accuracy, Precision, Recall, and F1_score) based on a Bayesian algorithm are between 83% and 86%. While this suggests a certain level of classification capability, its relatively lower performance indicates limitations in capturing complex intrusion patterns for real-time AVTP data. In contrast, the decision tree-based algorithm achieves improved results, with four evaluation indicators consistently falling between 91% and 93%. However, despite the performance gain compared to the Bayesian algorithm, it still leaves room for enhancement. The weighted histogram algorithm achieves an exceedance of 99% across all four evaluation indicators, demonstrating not only superior detection accuracy but also excellent balance among evaluation indicators. The weighted histogram algorithm’s substantial improvement reflects the ability to capture the nuanced features of AV data, enabling it effectively to differentiate between normal and abnormal communication behaviors. The findings clearly demonstrate that the weighted histogram algorithm developed in this study significantly enhances network intrusion detection performance when compared to traditional algorithms. Specifically, it achieves an improvement of 15.8% in detection rates over the Bayesian algorithm and 6.9% over the decision tree algorithm. This notable enhancement can be attributed to the algorithm’s ability to effectively capture and represent subtle variations in AV data through network intrusion detection architecture, which traditional probabilistic and rule-based classifiers fail to detect with comparable precision.

Moreover, the weighted histogram algorithm exhibits remarkable computational efficiency. Compared with Bayesian and decision tree algorithms, the proposed algorithm reduces processing time by 64.9% and 28.9%, respectively. This efficiency gain is particularly valuable in real-time in-vehicle communication environments, where timely intrusion detection is crucial for maintaining system stability and safety.

Computation time is a key indicator for evaluating computational complexity and practical deployment feasibility of intrusion detection algorithms, especially in real-time in-vehicle communication environments where latency and system responsiveness are critical. From the computational complexity perspective, Bayesian algorithms require probability estimation of features, involving the computation of conditional probabilities for each feature across all classes, which becomes increasingly time-consuming and memory-intensive as the number of features increases. In addition, the need to maintain and update the probability in the presence of streaming data introduces further computational overhead. Similarly, decision tree algorithms require repetitive partitioning of the feature space through recursive segmentation, which involves calculating impurity measures, selecting the optimal segmentation point, and constructing a hierarchical tree structure. These processes significantly increase the computational burden when the feature dimension is high or the data volume is large. In contrast, the proposed weighted histogram algorithm employs byte feature extraction with a sliding window mechanism to extract byte features of fixed length from each AV data, and then applies a sliding window mechanism to segment the incoming data stream into manageable time units. Within each window, a weighted histogram is dynamically constructed based on the frequency and importance of field values without complex probabilistic modeling or recursive tree construction. The whole process significantly reduces the computational overhead of single-round detection.

The weighted histogram algorithm also exhibits superior efficiency from the perspective of resource requirement utilization. Instead of storing and processing the entire data stream, it only needs to make a judgment according to the state of data within the sliding window to complete the dynamic update of the data frame. This allows for fast updates and efficient memory usage.

As a result, the weighted histogram algorithm not only ensures timely detection but also agrees well with the stringent performance and hardware constraints typical of IVNs. The algorithm achieves a balance between security and detection efficiency, providing a highly efficient network intrusion detection scheme for network security of In-Vehicle Ethernet.

## 5. Conclusions

This article focuses on the network intrusion detection problem of In-Vehicle Ethernet and proposes an innovative intrusion detection method based on a weighted histogram algorithm. In the large-scale and complex IVNs environment, the weighted histogram algorithm demonstrates significant advantages in terms of detection performance, real-time processing, and stability. The weighted histogram algorithm achieves an accuracy rate of 99.7% in detecting abnormal behaviors. In addition, the detection time of the weighted histogram algorithm is shorter than traditional intrusion detection algorithms, which satisfies the real-time requirements of In-Vehicle Ethernet. Future research work will focus on further exploring the network security issues of In-Vehicle Ethernet, along with constructing an integrated comprehensive protection system for intrusion detection, active prevention, and intelligent response, thereby promoting the secure development of In-Vehicle Ethernet technology and ensuring the network security of ICV.

## Figures and Tables

**Table 1:** Dataset of In-Vehicle Ethernet AVTP.

Dataset	Normal (Packets)	Abnormal (Packets)	Size (KB)
ROD	307,020	130,906	196,452
Label	0	36	16

**Table 2:** Comparison of network intrusion detection performance.

Algorithm	Accuracy	Precision	Recall	F1_Score	Time (s)
Bayesian algorithm	0.861	0.832	0.841	0.836	87.436
Decision tree algorithm	0.933	0.919	0.923	0.921	43.182
Weighted histogram algorithm	0.997	0.993	0.989	0.991	30.709

**Table 3:** The optimal solution parameter combination for the adaptive optimization module.

Parameter Name	Parameter Optimal Value
Feature sampling rate $s_{1}$	0.584
Classification node number $s_{2}$	28
Maximum classification frame rate $s_{3}$	9

## Data Availability

The data that support the findings of this study are not publicly available due to privacy concerns. Requests for access to the data should be directed to the corresponding author.

## Abbreviations

The following abbreviations are used in this manuscript:

ICV	Intelligent Connected Vehicle
IVNs	In-Vehicle Networks
LiDAR	Light Detection and Ranging
ECUs	Electronic Control Units
ADAS	Advanced Driver Assistance Systems
IVI	In-Vehicle Infotainment
AVTP	IEEE 1722 Audio Video Transport Protocol
TSN	Time-Sensitive Networking
LIN	Local Interconnect Network
CAN	Controller Area Network
IDS	Intrusion Detection Systems
MAC	Message Authentication Code
AV	Audio and Video
ID	Identifier
WiFi	Wireless Fidelity
DoS	Denial of Service
UDP	User Datagram Protocol
CAN/FD	Flexible Data-Rate
AVTPDUs	AVTP Data Units
ROD	Randomly Obtain Dataset
TP	True Positive
TN	True Negative
FP	False Positive
FN	False Negative
